# The inner clock—Blue light sets the human rhythm

**DOI:** 10.1002/jbio.201900102

**Published:** 2019-09-02

**Authors:** Siegfried Wahl, Moritz Engelhardt, Patrick Schaupp, Christian Lappe, Iliya V. Ivanov

**Affiliations:** ^1^ Institute for Ophthalmic Research University of Tuebingen Tuebingen Germany; ^2^ Carl Zeiss Vision International GmbH Aalen Germany

**Keywords:** visible light, blue light, circadian rhythm, melanopsin, melatonin

## Abstract

Visible light synchronizes the human biological clock in the suprachiasmatic nuclei of the hypothalamus to the solar 24‐hour cycle. Short wavelengths, perceived as blue color, are the strongest synchronizing agent for the circadian system that keeps most biological and psychological rhythms internally synchronized. Circadian rhythm is important for optimum function of organisms and circadian sleep–wake disruptions or chronic misalignment often may lead to psychiatric and neurodegenerative illness. The beneficial effect on circadian synchronization, sleep quality, mood, and cognitive performance depends not only on the light spectral composition but also on the timing of exposure and its intensity. Exposure to blue light during the day is important to suppress melatonin secretion, the hormone that is produced by the pineal gland and plays crucial role in circadian rhythm entrainment. While the exposure to blue is important for keeping organism's wellbeing, alertness, and cognitive performance during the day, chronic exposure to low‐intensity blue light directly before bedtime, may have serious implications on sleep quality, circadian phase and cycle durations. This rises inevitably the need for solutions to improve wellbeing, alertness, and cognitive performance in today's modern society where exposure to blue light emitting devices is ever increasing.

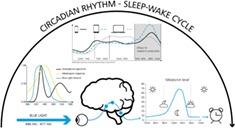

## Graphical abstract

Light is playing an important role beyond vision. The review summarizes the current and most recent knowledge on the role of light around 470 nm and its role in circadian rhythm. The circadian rhythm is essential for life. If it is not preserved, serious diseases might develop. Light and its role in synchronization, but also in disruption of the circadian rhythm is discussed.

## 1 THE ADVENT OF THE CIRCADIAN PHOTORECEPTOR

**Figure 1:**
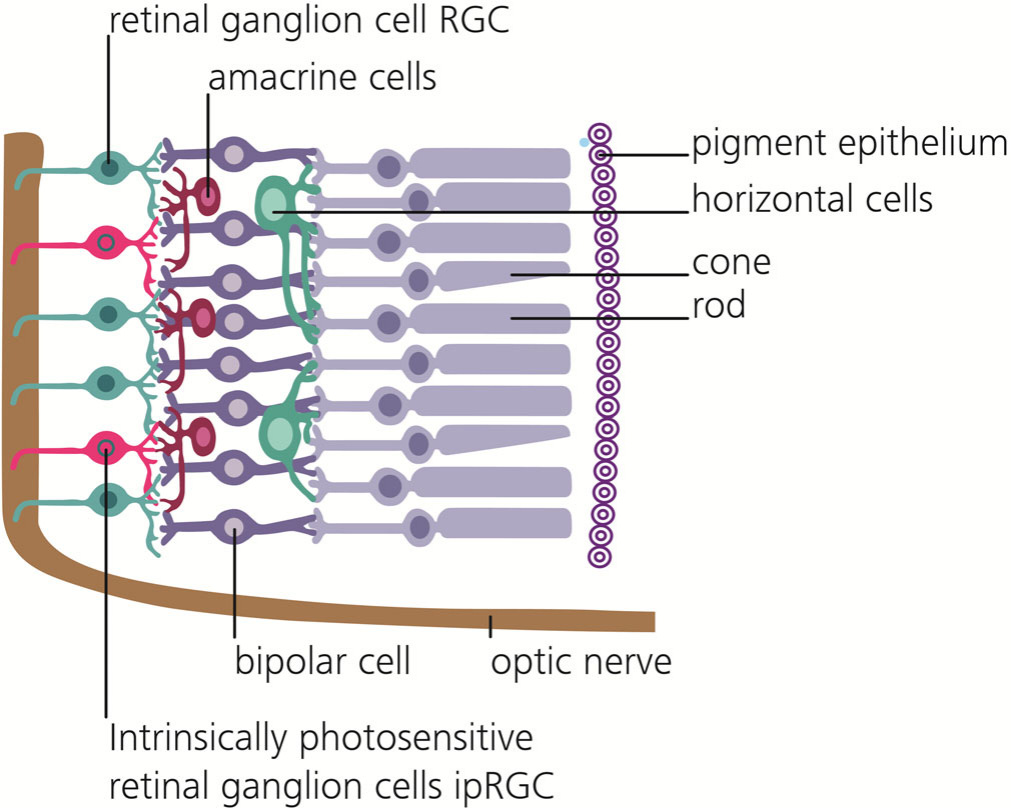
Cross sectional view of the retinal system. Light traverses the system from the left, cones and rods transmit visual information via the bipolar cells, amacrine cells, and ganglion cells to the optic nerve. The sparse subset of intrinsic photosensitive retinal ganglion cells can induce signals themselves, due to their possession of a separate photopigment, melanopsin

**Figure 2:**
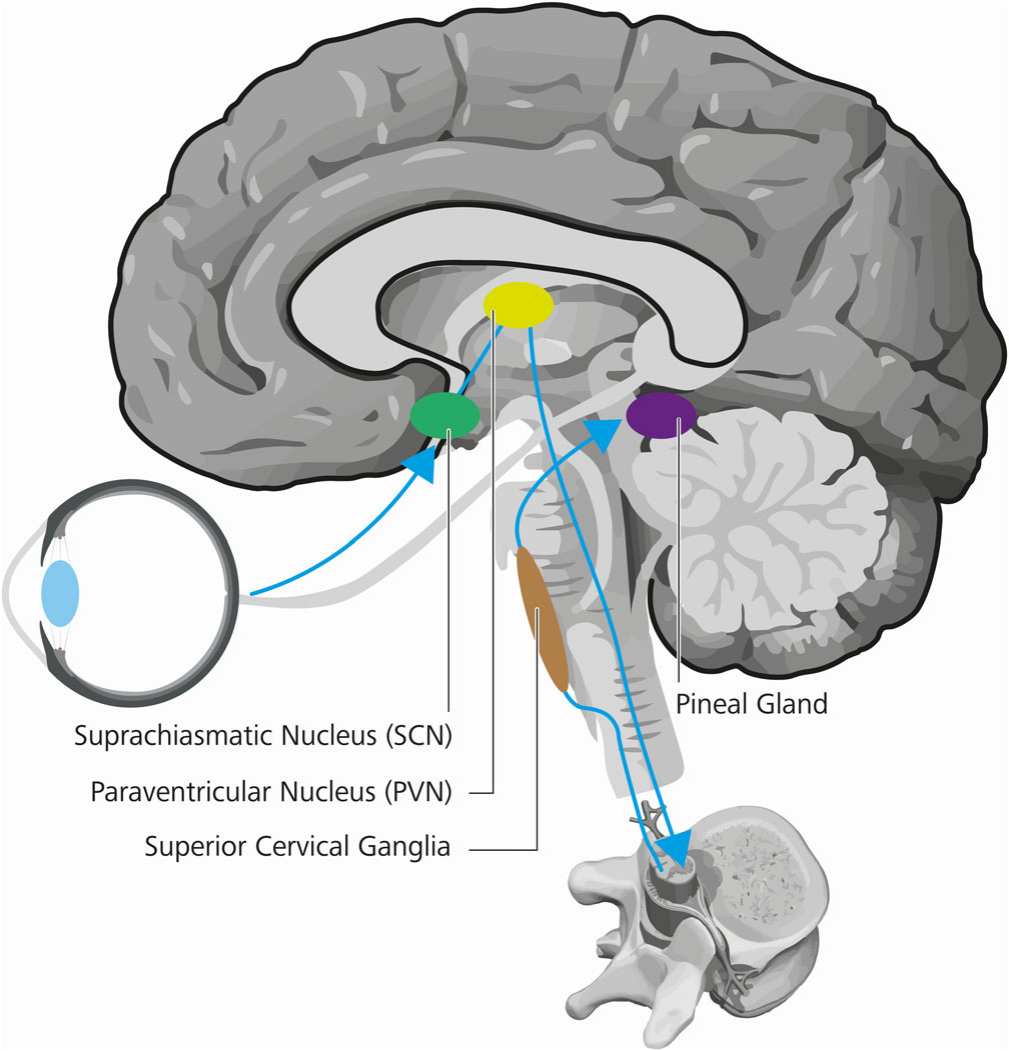
Signal transduction of circadian information. A melanopsin induced signal from the ipRGCs is transmitted via the retino‐hypothalamic tract (blue) to the hypothalamic pacemaker neurons in the suprachiasmatic nucleus (green), the human “master clock”. The circadian information is transmitted further downstream via the paraventricular nucleus (yellow), intermediolateral cell column in the vertebral gray matter, superior cervical ganglion (brown) to the pineal gland (purple), which is responsible for melatonin secretion

Circadian rhythms are biological cycles that have about 24‐hour periods. Body temperature, hormonal levels, sleep duration and quality, cognitive performance and countless other physiological variables exhibit such daily oscillations. The ability of the visible light to synchronize the biological clock of the photosynthetic marine dinoflagellate *Gonyaulax polyedra* to the solar 24‐hour cycle is known since 1958 1. It was not until 1998, when a new type of photoreceptor in the human eye was discovered 2 and later identified to be especially sensitive to blue light. Importantly, these new photoreceptors are retinal ganglion cells (RGCs) and communicate directly with the brain. The melanopsin containing retinal ganglion cells, called intrinsic photosensitive retinal ganglion cells (ipRGCs) count for around 1% of the total RGCs and are found in front of the retina (see Figure 1) to process and send signals 3, 4, 5 among others to the suprachiasmatic nucleus (SCN), the so called master clock of the brain 6. In depth morphological analyses allowed for an ipRGC population clustering into five distinct subtypes, which moreover express a certain diversity in their dendritic stratification, morphology, membrane properties, and melanopsin expression levels 7. Worth mentioning are hereby the most light sensitive M1 cells, which in turn can be separated according to their Brn3b expression, with Brn3b‐negative cells operating as the herein described reporters to the SCN, but likewise signal to the olivary pretectal nucleus to influence the pupillary light reflex 7. Other, less light sensitive, subtypes project to further, even image forming, brain areas, indicating potentially different functional responsibilities, thereby influencing many aspects of human physiology 7. These circadian photoreception cells differ from the classical rod and cone photoreceptors in many ways. They use a unique photopigment, melanopsin and have lower sensitivity and spatiotemporal resolution than rods or cones. Light depolarizes ipRGCs but hyperpolarizes rods and cones. Further, ipRGCs are less sensitive than the classical photoreceptors and are far more sluggish, with response latencies as long as 1 minute. Their strongly overlapping dendrites, which are themselves filled with melanopsin, are able to respond directly to light 4, which is why the entire ipRGC population has been referred to as” photoreceptive net”^8^. Bright continuous illumination evokes a sustained depolarization in ipRGCs that encodes stimulus energy. This sets these cells apart from all other photoreceptors, which are less sensitive to encode irradiance and thus to represent ambient light intensity 5, 9, 10 over days 11 and months 12. These properties are well suited to nondirectional detection of gross environmental illumination essential for integrated circadian, neuroendocrine and neurobehavioral effects 5. They also contribute to the pupillary light reflex and other behavioral and physiological responses to environmental illumination. The M1 Brn3b‐negative ipRGC photoreceptors send unconscious, nonvisual photic information through the retinohypothalamic tract to the SCN (see Figure 2) permitting alignment of internal biological with external environmental time. Cells in the SCN thereby create a circadian rhythm via interconnected gene‐protein feedback loops, wherein transcription and activity of clock genes and transcribed proteins function as their own regulators 13. Worth mentioning is the control loop including Period (Per1, Per2, and Per3) and Cryptochrome (Cry1 and Cry2) genes, whose transcription is promoted by transcription factors Circadian Locomotor Output Cycles Kaput (CLOCK) and Brain and Muscle ARNT‐like protein 1 (Bmal1), whose transcribed proteins Per & Cry in turn inhibit CLOCK/Bmal1‐mediated transcription 13. These feedback‐loops in turn require external cues, among others light 4, social interactions 14 and nutrition 15, to maintain a 24‐hour schedule 13. The resulting SCN pacemaker neuron activity subsequently synchronizes the rhythms in subsidiary clocks of peripheral tissue, which would otherwise run free and desynchronize 16. Therefore, although absent or deficient ipRGC photoreception cannot be perceived subjectively 17, the missing entrainment yields in circadian disturbances, which can have significant physiological and psychological consequences 18.

## 2 IMPORTANCE OF BLUE LIGHT IN REGULATING HUMAN BEHAVIOR AND CIRCADIAN RHYTHM

**Figure 3:**
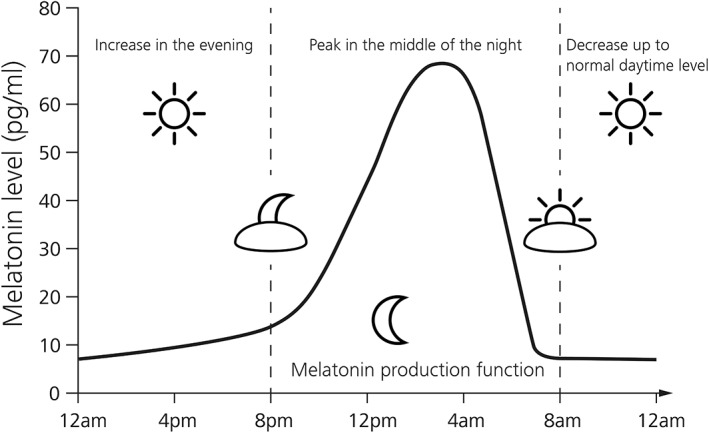
Physiological melatonin levels of a 24 hour day. Declining light exposure in the evening results in an increased melatonin production by the pineal gland with peak levels in the middle of the night almost 10‐fold higher than regular day levels (adapted from 33)

Cones, the color receptors of the human visual system, are classified according to the wavelength sensitivity peak of their respective photopigments, wherefore they are commonly referred to as short (S, 420 nm), medium (M, 533 nm), and long (L, 562 nm) wavelength cones 19. Under photopic conditions, these spectral opsin sensitivities give rise to the photopic luminosity function, describing the relative sensitivity of the human eye to white light intensities, with a peak around 555 nm 20. Rods on the other hand only rely on one photo pigment, rhodopsin, which is most sensitive at 498 nm 19. Due to their retinal ganglion cell nature, ipRGCs receive, besides their own melanopsin based photosensitivity, intraretinal synaptic input from classical image‐forming photoreceptor driven circuits 21. This input potentially modulates the nonimage‐forming ipRGC light response transmitted to higher brain regions 21. The extrinsic photoreceptor influence is most notably light intensity dependent, whereby rods impact the intrinsic ipRGC response primarily in scotopic conditions (~7 log photons cm^−2^ s^−1^), while cones contribute in photopic conditions (~11 log photons cm^−2^ s^−1^), although less pronounced 22, 23. Taking this photoreceptor intensity sensitivity, the melanopsin phototransduction threshold (~9 log photons cm^−2^ s^−1^) and the respective dynamic ranges into account, an ipRGC is able to encode light intensities in a range of 9 orders of magnitude, with modestly varying spectral sensitivities as a results of extrinsic photoreceptor input 24. Sensitivity of human nocturnal melatonin suppression 25, 26 peaks in the blue portion of the light spectrum around 460 nm, most effective in the range between 446 and 477 nm, manifesting a substantial contribution of short wavelength visible light not directly corresponding with an established single opsin maximum sensitivity 27. Spectral absorption by melanopsin 28, which is the photopigment expressed in cell bodies and elongated dendrites 5 of ipRGCs, peaks at 479 nm 29. The difference of ~20 nm might reflect an influence of short wavelength cones on the melatonin response. Thus, blue wavelengths can potentially exert more powerful effects on human circadian rhythm than green and yellow wavelengths. This possibility has been confirmed experimentally 30 when suppression of melatonin in humans was compared during nighttime exposure for monochromatic light at 460 nm with light at 555 nm, the peak sensitivity of the visual cones. The blue wavelength suppressed melatonin for about twice as long as green, despite the equal photon density. Blue monochromatic light exposure was more effective to cause a phase delay of the circadian rhythm of all subjects, which were studied in an environment free of time cues. Circadian phase assessments were done by monitoring the melatonin secretion profiles (see Figure 3 for a physiological profile), before and after monochromatic light exposure (6.5 hours), while subjects performed constant routines. While the photon flux was matched for different wavelengths, the observed effect that blue light was more effective in circadian shifts suggests that ipRGCs do not simply count or average photons but are rather sensitive to the particular wavelength of exposure 30. Similar wavelengths also proved more powerful in elevating body temperature and heart rate and in reducing sleepiness, while provoking responses in alertness‐related subcortical structures and limbic areas, which established light, most notably in the blue range, as a potent modulator of brain function 31, 32.

Evidence for the importance of light in regulating the circadian rhythm also come from studies that used bright light to treat mood disorders 34. Melatonin suppression in humans was observed by applying bright light of 2500 lx, much brighter than indoor lighting, but much less so than a cloudy day. Therefore, it was concluded that the circadian rhythm is best cued by natural bright light and would be insensitive to dim indoor lighting. The finding that bright light can suppress melatonin 34 led to treating seasonal affective disorder with bright light. The literature on the efficacy of blue light for treating this disorder is just now beginning to develop, but research on the efficacy of blue light to suppress melatonin suggests that exposure to blue light might be more effective treatment. Blue light may further prove useful for treating premenstrual depression, bulimia, and anxiety 32. Personalized blue light exposure raised activity levels during daytime hours and increasing sleep at nighttime in a long‐term care facility for patients with Alzheimer's disease and related dementia 35. An investigation of dysfunction‐pathologic structure relationships of the hypothalamus in Alzheimer's disease patients could likewise prove a direct effect of light therapy on circadian rhythm related disturbances 36.

Further, a correlation of polychromatic light intensity and nocturnal melatonin suppression could be demonstrated, with 1 hour of 1000 lx exposure by night being sufficient to reduce melatonin levels to a daytime average 37. Studies indicate that low levels of illumination are sufficient to trigger awakening and there are hints that the effect on circadian rhythm gets saturated at high illumination levels 38, 39. Electroencephalography has shown that light exposure influences alpha, theta, and low‐frequency activity, which are correlates of sleepiness 31. Blue light proved superior to other wavelengths in enhancing responses in the left frontal and parietal cortices during a working memory task. Experimental subjects had quicker auditory reaction times and fewer lapses of attention under blue light than green light 31. Blue wavelengths suppressed sleep‐associated delta brainwaves better than green wavelengths and boosted the alpha wavelengths, which are related to alertness 31. This suggests that short wavelengths, perceived as blue color, might be used to control sleepiness. Therefore, blue light seems to be the strongest synchronizing agent for the circadian system that keeps most biological and psychological rhythms internally synchronized. The sleep/wake cycle is however not to be equated with circadian rhythms, since additional factors, most notably the level of homeostatic sleep drive, which only diminishes by sleep itself, affect sleep propensity. The expression of circadian rhythms nevertheless seems to be most obvious in the rhythmicity of sleep and neurobehavioral performance in humans 40. It has further been hypothesized that light has additional, noncircadian effects on alertness and the sleep/wake cycle, most notably by acting on sleep promoting, as the ventrolateral preoptic nucleus, and wake promoting brain regions either directly or eventually via the SCN 41. These conclusions reached further, as to suggest, that rod and cone encoded light stimuli were primarily involved in the promotion of the wake state, whereas melanopsin primarily evokes a higher level of alertness by mediating EEG correlates of waking associated cognitive processes 41. It is recognized, that subjective alertness is moreover influenced by subjective aspects, such as well‐being and visual comfort 42. The power to reset the organism's daily meter declines into green (505 nm) 43 and green‐yellow (555 nm) colors 30. Thus, today it is understood that blue light has many physiologic effects but perhaps the most important among them is to entrain the circadian rhythm 5. A selection of studies, investigating the effects and details of spectral composition, intensity, duration, timing and temporal distribution of light stimuli 44 on human behavior and circadian rhythm are outlined in Table 1 of the appendix.

**Table 1:** Selective overview of published studies and methodological parameters regarding the effect of light, most notably in the blue spectrum, and illumination intensity on circadian rhythms, alertness, and sleep. Most studies in humans included an initial ophthalmological examination and evaluation of chronotype, constant posture protocols in the laboratory and dark adaptation episodes under polychromatic dim light for 0.5 to 2 hours before treatment exposure. Irradiance unit summarizes information about photon density, irradiance, illuminance, and luminance. The properties timing and duration refer to start and length of the respective treatment exposure, not the whole experimental protocol

		Light parameter					
Study	Methodology	Spectral composition	Unit	Duration	Timing	Subjects (mean age ± SD)	Results
Lockley et al 30	BMA	555 and 460 nm	6 × 10^13^ photons cm^−2^ s^−1^	6.5 h	9.25 h before waketime	16 healthy, (23.3 ± 2.4)	460 nm induced a 2‐fold greater circadian phase delay
McIntyre et al 37	BMA	polychromatic	‐	1 h	Midnight	13 healthy, (25.1 ± 6.4)	1000 lx intensity suppress melatonin to near daytime levels; 350 lx significantly suppress nocturnal melatonin levels.
Munch et al 43	Sleep: PSG, 8 EEG, EOG, EMG, ECG	550 nm, 460 nm, polychromatic	460 nm: 12.1 μW cm^−2^, 550 nm: 10.05 μW cm^−2^	2 h	9:30 PM	8 healthy, male (24.6 ± 3)	Small wavelength‐dependent effects of light on sleep architecture and EEG. Most likely an acute alerting effect continuing into sleep and/or an immediate phase delay induced by blue light.
Herljevic et al 48	BMA	548 and 456 nm	7000‐12 000 lx vs. 150 lx	0.5 h	˜3.5 h pre melatonin peak	13 pre‐ and 21 post‐menopausal women	Reduced melatonin suppression in elderly subjects following exposure to blue light, likely due to age‐related changes in lens density.
Sletten et al 49	Questionaires: Alertness, sleepiness, mood, BMA	548 and 456 nm	200‐400 lx	2 h	8.5 h after DLMO	11 young (23.0 ± 2.9), 15 old (65.8y ± 5.0) men.	Subjective alterness, sleepiness and mood response to blue light diminished in elderly compared to young subjects. No age effect in green light exposure.
Cajochen et al 57	KSQ, CBT and surface skin temperature, ECG, SMA	550 nm, 460 nm, polychromatic	2.8 × 10^13^ cm^−2^ s^−1^	2 h	10 h after waketime	10 healthy, male (25.9 ± 3.8)	Blue light exposure in the evening suppressed melatonin more than green light exposure, and effected increased alerting response, core body temperature and heart rate.
Czeisler et al 59	Night shift work treatment; Sleep‐wake logs, ECG, CBT, Cognitive‐performance tasks	Polychromatic	150 and 7000‐12 000 lx	8 h	Midnight	8 healthy, male, 22‐29 years	Maladaptation of the human circadian system to night work can be treated with scheduled exposure to bright light at night and darkness during the day.
Davidson et al 62	Molecular rhythms, high‐resolution optical microscopy & bioluminescence of SCN	Polychromatic	200‐400 lx	6 h phase shift		mPer2LUC knock‐in mice	Differences in circadian shifting kinetics are apparent among subjects and among organs.
Khalsa et al 63	BMA	Polychromatic	fixed gaze ∼10000 lx; free gaze ~5000‐9000 lx	6.7 h, alternating gaze	centered in 16 h wake episode	21 healthy, entrained	Phase delays occur when light stimulus is centered prior to the core body temperature minimum (CBTM), phase advances occur when light stimulus is centered after the CBTM, no phase shift occurs at CBTM.
Cajochen et al 68	SMA, KSQ, KDT, GO/NOGO task, cognitive performance	LED with twice more 464 nm emission	LED 0.241 Wsr^−1^ m^−2^; CCFL 0.099 W sr^−1^ m^−2^)	5 h	6 h before bed time	13 male (23.8 ± 5.0)	Evening exposure to blue enriched LED screen resulted in attenuated salivary melatonin and sleepiness levels, accompanied with increase in cognitive performance.
Wahnschaffe et al 71	Sleep log and actimetry; SMA, VAS	White, zero blue component, high intensity light	130 and 500 lx	0.5 h	1 h before bedtime	9 healthy, (26.3 ± 4.2)	Yellow light exposure did not alter the increase of melatonin in saliva. Lighting conditions including blue components reduced melatonin increase significantly. Subjective alertness was significantly increased after exposure to light which included blue spectral components.
Chang et al 73	Blood plasma melatonin assay, PSG, KSQ, EEG	LE‐eBook: blue enriched; Print book: white light	30 and 3 lx	4 h	6 pm	12 healthy, (24.9 ± 2.9)	LE‐e‐book condition compared to print book: suppressed evening levels of melatonin, delayed melatonin onset, delay sleep onset, less rapid eye movement (REM) sleep.
Kozaki et al 74	SMA at 1 am and 9 am	Polychromatic white & blue enriched light	300 and 10 lx	1.5 h	1 am, 9 am	12 healthy, male (21.9 ± 0.9)	Findings suggest that daytime blue light exposure has an acute preventive impact on light‐induced melatonin suppression.
Kervezee et al 98	Night shift protocol: transcriptome assay	Polychromatic	mean 2.6 ± 0.4 lx (SD)	8 h	10 h phase shift	8 healthy	Reduction of rhythmic transcripts in the night shift condition. Mainly due to dampened rhythms & lower amplitudes rather than to a complete loss of rhythmicity.

Abbreviations: BMA, blood melatonin assay; CBT, continuous body core temperature; DMLO, dim light melatonin onset; ECG, electrocardiogram; EEG, electroencephalography; EMG, electromyogramm; EOG, electrooculography; KDT, Karolinska drowsiness test; KSQ, Karolinska sleep questionnaire; PSG, polysomnography; PVT, psychomotor vigilance task; SMA, saliva melatonin assay; VSA, visual analogue scale.

## 3 BLUE LIGHT IMPACT ON AGING AND RETINAL DAMAGE

Age‐related changes in lens density 45, 46 are known to reduce the transmission of blue light, which has been shown to be most effective in suppressing melatonin secretion during the day. A further age‐related change in the eye that may contribute to reduced levels of light reaching the ipRGCs is the reduction in pupil size 47. Therefore, this diminished blue light input to the circadian clock may result in disturbed circadian rhythm and sleep in the elderly. Age‐related changes in melatonin suppression were first studied by Herljevic 48, where suppression was compared across light conditions and between age groups. Two groups of young and elderly subjects were exposed for 30 minutes to blue and medium wavelength light at different irradiances. Light exposure was adjusted so that each subject received light on the rising phase of its endogenous melatonin rhythm. Significantly reduced melatonin suppression was measured in the elderly subjects in response to exposure to blue (456 nm) light as compared to the young group. This finding is suggested to reflect the age‐related changes in lens density. In another study 49, it was further demonstrated that similar age‐related reduction occurs in subjective alertness, sleepiness, and mood following blue light exposure. The reduction in responsiveness to the effects of blue light in older people was not observed following green light exposure. The magnitude of circadian phase advance was assessed by monitoring the difference in plasma melatonin rhythm before and after light exposure 49. The phase advances to both blue and green light were larger in the young than older subjects, although differences did not reach statistical significance. To analyze how age‐related losses in crystalline lens transmittance and pupillary area affect circadian rhythm phakic and pseudophakic individuals of the same age range were studied 50. In phakic eyes the natural lens is left untouched after intraocular lens (IOL) implantation to correct myopia, while in pseudophakic eyes the natural lens is explanted. The age‐related decline in retinal illumination was estimated taking into account human crystalline lens transmittance at different ages and the pupil area for those ages. Pupil‐weighted spectral retinal illumination was multiplied wavelength by wavelength with melatonin suppression sensitivity between 350 and 700 nm to determine how aging affects circadian photoreception for different monochromatic lights. Results showed a 10‐fold greater circadian photoreception in young children (10 years old) than adults (95 years old) with phakic eyes. Pseudophakia improves circadian photoreception at all ages, particularly with UV‐only blocking IOLs that transmit blue wavelengths. A recent study further suggests that the aging of retinal tissue most severely effects the reduction in amplitude of rhythm of the circadian clock 51.

### 3.1 More facets of blue light

**Figure 4:**
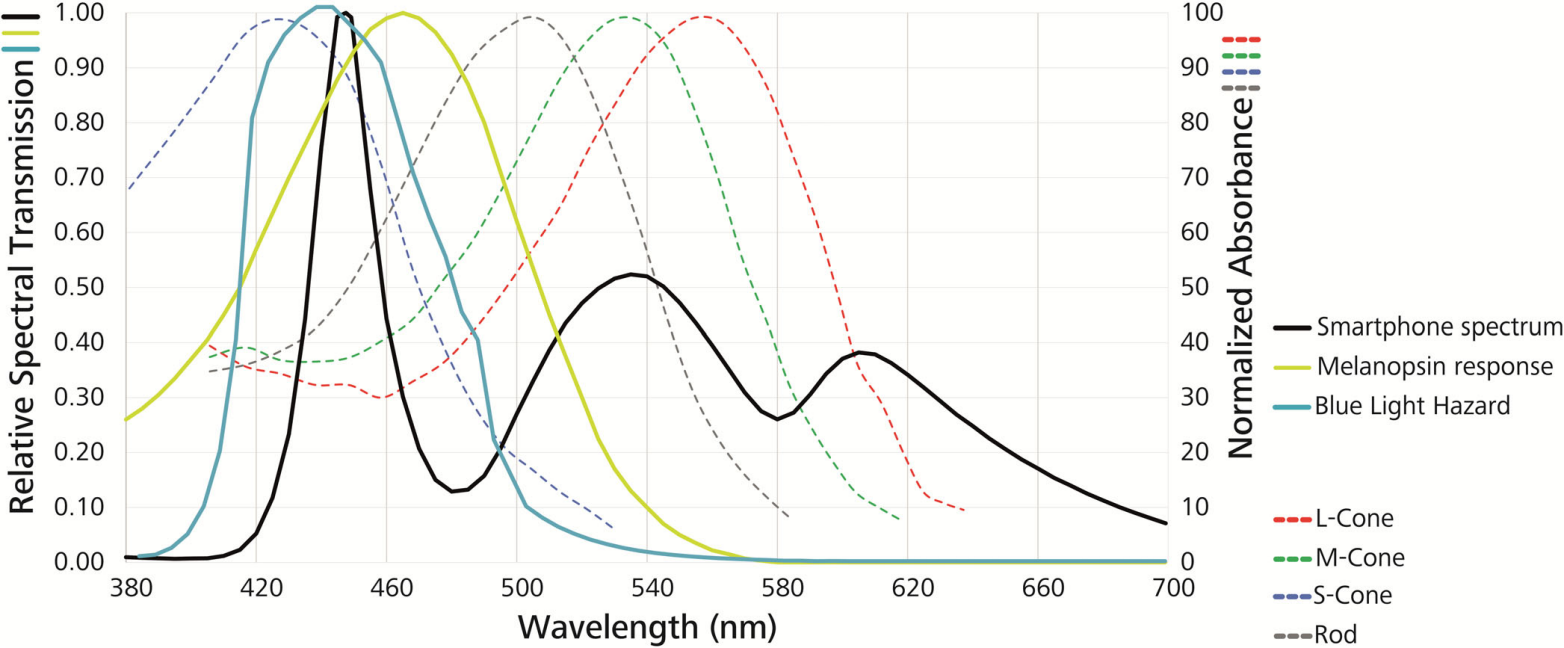
Irradiance of the blue light hazard function. The Blue light hazard function according to ISO 8980 represents the relative spectral sensitivity of the human eye to blue light hazards, based on the effectiveness of radiation to induce photoreversal of bleaching. The emission spectrum of an ordinary smartphone screen on maximum brightness shows a distinct overlap in the potentially harmful blue peak area, and melanopsin sensitivity, but also image‐forming related S‐cone and rod absorbance spectrum. Normalized absorbance spectra are depicted according to Bowmaker and Dartnall, 1980 19

While short wavelengths in the range of 460 nm are very efficient in phase shifting of circadian system, intensive blue light in the range between 400 and 440 nm (see Figure 4) is damaging to the retina via a photochemical reaction called photoreversal of bleaching 52. It augments the capability of rhodopsin molecules to absorb photons by several orders of magnitude, thus allowing the molecules to reach the critical number of photons required to induce damage in the retinal cells. This process can further increase the potential production of reactive oxygen species (ROS). The oxidative damage can lead to the accumulation and build‐up of lipofuscin in the retinal pigment epithelium (RPE). The build‐up of lipofuscin in the RPE can affect its ability to provide nutrients to the photoreceptors, affecting their viability. The severity of the blue‐light induced damage depends on the time of the day and is thus related with the circadian rhythm 52. Accumulation of ROS and lipofuscin may be due to both acute and cumulative exposure to blue light 52. While the mechanisms behind the effect of long‐term sub‐threshold exposure to blue light are not fully understood, it seems to be linked to the lipofuscin level in RPE 52. Lipofuscin is mainly formed in the photoreceptor outer segments as byproduct of the degradation of the disks. When it absorbs blue light, ROS are produced. The type of ROS created is related to the specific wavelength (*λ*) of the blue light. The amount of lipofuscin in the retina increases with age and is related to age‐related macular degeneration 52. However, the detrimental effect of blue light on the retina should be taken with caution. Most of the damaging effects through blue light are demonstrated in nocturnal animals only, or in isolated animal cell cultures directly exposed to blue light 52, 53, 54. In short, animal and in vitro experiments suggests some evidence for photochemical retinal damage upon acute blue light exposure. In humans, a direct evidence of acute light‐induced damages to the retina from accidental high‐intensity artificial or sunlight exposure is apparent. Ultimately, there is no consistent evidence that long‐term exposure to blue light at lower intensity causes any damage to the retina 55. For even shorter wavelength, ultraviolet radiation, the evidence is more clear 56.

## 4 THE TIMING OF BLUE LIGHT AND CIRCADIAN DISRUPTION

Circadian rhythms, including melatonin rhythms, are involved in different aspects of facilitation of sleep 57, 58. Chronic light exposure at the wrong time, at night during shift work for example, may contribute to shifts of the circadian clock phase 59, dependent on duration, wavelength, and intensity of light 60. While suppressing melatonin secretion during the day is believed to be beneficial, it is not so at night. Circadian disruption is mainly characterized by misalignment between the internal circadian rhythms and the external environmental conditions. Circadian disruption also includes asynchronous SCN with the sleep cycle and with peripheral oscillators in tissues throughout the body, since these peripheral clocks adapt unevenly depending on the tissue 16. So far, these peripheral circadian clocks, and in some instances their intrinsic zeitgebers, could be identified in almost all investigated mammalian cells 61. A circadian desynchronization will persist for a variable period of time depending on the exposure pattern and the characteristics of the individual 62, including age and chronotype: morning or evening birds. Light exposure may induce phase advances or delays when applied at different times in the circadian cycle. For instance in humans, light between about 5 am to 5 pm advances, and light outside this interval delays the circadian clock 63. Therefore, chronic light exposures at the wrong time may induce phase shift of the circadian system, not allowing for synchronization to the external environmental conditions, and leading to circadian disruption, thus altering both cellular and organ function 61.

**Figure 5:**
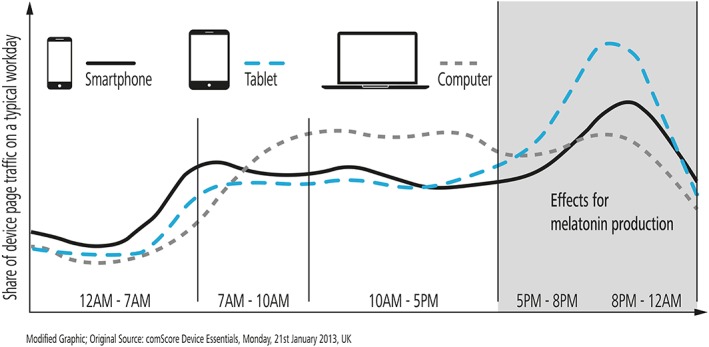
Light emitting device usage during the day. Usage has been approximated by the share of device dependent web page traffic. It has been reported, that blue light exposure up to 4 hours prior to sleep can affect the melatonin levels at night and subjective sleep quality. Figure modified according to 79

When the effects between exposures during the day to blue‐enriched white light and white light were compared, the blue‐enriched higher color temperature lamps significantly influenced sleep onset (earlier) and reduced sleep latency in the personnel at a research station in Antarctica 64. This result, suggesting that blue‐enriched white light synchronized the circadian rhythm, is corroborated with other results showing that blue light is more efficient in melatonin suppression than other wavelengths 65, 66, and can thus be seen as the most potent zeitgeber. Altered slow wave activity (sleep depth) at the end of the subsequent night of sleep indicated that circadian phase delays might be induced by light exposure before bedtime 43. These delays are persisting into sleep 43 with a greater effect of blue than green light, suggesting that blue light exposure before bed time can affect sleep. A comparison between the effects of living room light (less than 200 lux) and dim light (about 3 lx) before bedtime showed that even low levels of light in house settings may be sufficient for circadian disruptions in humans: Exposure to room light suppressed melatonin levels and shortened the duration of melatonin production in healthy young subjects 65. Dim (32 lx), blue depleted LED light, 4 to 7 hours after the subject's respective waketime appeared to impact the post‐illumination pupil response significantly less than regular white LED light, without affecting the melatonin level or subjective sleepiness 67. Evening exposure to a LED screen, with more than twice the level of blue light (460 nm) emission of a control screen, significantly lowered evening melatonin levels and suppressed sleepiness 68. For low light levels (40 lx), the melatonin suppression is significantly greater 69 after 2 hours of exposure in the evening to blue‐rich (6500 K, CFLs light) than to incandescent light (3000 K). A recent study by Cajochen et al 42 moreover reported, that the alerting effect of light sources with a prominent blue peak appeared to be stronger in the evening and night, compared to morning hours. This alerting effect‐exposure timing dependence has further been reported in the context of absent alerting responses during daytime light exposures, suggesting a relationship to circadian features, such as the melatonin level 70. In another experiment 71 exposing healthy young participants in their natural home environment to 30 minutes of 500 lx blue light an hour before bedtime delayed the onset of rapid eye movement (REM) sleep by 30 minutes. Melanopsin RGCs are extremely sensitive to blue light (see Figure 5) and even exposure to light levels as low as the one from a smart‐phone or light emitting e‐readers are associated with disruptions of circadian rhythm. Presence of smart‐phones in modern life is ever increasing and longer average screen times have been shown to result in shorter sleep duration and worse sleep efficiency 72. Reading a light emitting e‐book before sleep, as compared with printed book, increased the time to fall asleep 73 in young adults (25 ± 3 years old). In individuals reading e‐books before bed‐time, their circadian clock was delayed, assessed by delayed and reduced phase of rapid eye movement sleep. Melatonin blood concentration levels were suppressed and alertness on the next morning was reduced. Use of the light emitting e‐books immediately before bed time also increased alertness at that time. On the other side, daytime blue light exposure has an acute preventive impact on nocturnal light‐induced melatonin suppression 74. Similar tendencies, suggesting increased melatonin suppression and delayed circadian rhythm timing, decreased evening sleepiness and increased morning sleepiness, due to unrestricted light emitting device usage in the evening, have been reported recently 75. The preventive effect of blue daytime light has proven more efficient than other wavelength composition of daylight. The implications of these findings 65, 68, 69, 72, 73 are, that the melanopsin receptors are particularly sensitive during the evening and nighttime hours, and suggest that many of the sleep disorders may be related to low levels of blue light exposure in the evening, just before bed time. Beside the influence of irradiance it was also shown that color signals, particularly the blue‐yellow color discrimination, entrain the inner clock 76. Narrow band blue light is used today to treat problems such as sleep disorders, jet‐lag 77, seasonal affective disorder 78, and premenstrual syndrome. For this, ipRGC photoreceptors are stimulated via light emitting goggles, panels, and other devices.

## 5 CIRCADIAN DISRUPTION AND ILLNESS

Chronic disruptions of circadian rhythm may have the potential to seriously affect human health. For instance, decrease of melatonin levels plays an important role in development of chronic diseases and conditions such as cancer 61, 80, 81, 82, 83, cardiovascular diseases 83, reproduction 84, endometriosis 84, gastrointestinal and digestive problems 85, diabetes 86, 87, obesity 88, depression 89, sleep deprivation 90, bipolar spectrum disorders 91, and cognitive impairment 92. However, it is extremely difficult to directly expose the effects of wrong timed light exposures and their long term health consequences, since in modern society expose to artificial lighting is virtually unavoidable. Therefore, epidemiologic studies that suggest light involvement in health risk 93, 94 mainly provide indirect evidence. In general, it is suggested that circadian disruptions are increasing health risk in night‐shift workers and flight attendants potentially suffering from both jet‐lag and night‐shift work. While presence of ambient light during the night sleep is related with circadian disruptions, a decreased health risk is related with long sleep duration and in blind women 95. Further, results from prospective cohort studies consistently suggest that women with the lowest concentration of the main melatonin metabolite sulfatoxymelatonin, have the highest risk for breast cancer 96, 97. A recent study could link night shift work with a loss in temporal coordination between the human circadian transcriptome and its environment, most notably by affecting the natural killer cell‐mediated immune response and Jun/AP1 and STAT pathways 98.

The most convincing evidence of an association between circadian disruption and health risk is found for breast cancer in night‐shift workers. Long periods of night‐shift work, which may occur for several years, affects about 10% to 20% of the EU workforce. Prolonged periods of work during the night are the most extreme source of wrong timed exposure to light, which leads to a simultaneous reduction of melatonin production, sleep deprivation and circadian disruption 99. A recent meta‐analysis 100, adjusted for potential confounders in published studies of shift work and female breast cancer risk, demonstrated a significantly increased risk of 40%. Further evidence for the association between shift work and breast cancer 101, 102, 103, 104, 105 point toward exposure to light at night as causal factor, but see 106. A significant association between breast cancer risk and exposure to nonoccupational light during the night at home is found for women who did not sleep during the period of melatonin level peaks, or who frequently turned on the light during the night 107, 108, 109. Similarly, increased breast cancer risk is also correlated with increasing bedroom light levels. These results are based on self‐reports of light exposure and therefore prone to recall bias, which may limit interpretations. Based on evidence in animal experiments on the carcinogenicity of light during biological night, and evidence from epidemiologic studies in humans on the carcinogenicity of light exposure during night work, shift work that involves circadian disruption has been recognized by the International Agency for Research on Cancer (IARC) as probably carcinogenic to humans, Group 2A 82, 110.

It is further hypothesized, that the SCN needs repeated input by external, as well as metabolic factors to sustain synchronization between endogenous physiological rhythms and external demands. Modern, hectic lifestyles with omnipresent and energy efficient lightning pollution often lead to a desensitization of the biological clock which results in abnormal endocrine responses, a basic component of type 2 diabetes 87.

Investigations into the cardiovascular system revealed diurnal variations in gene expression, protein expression, and organ function 111 and a potential influence of both inter‐ and intra‐organ circadian clock disruptions on the pathogenesis of the cardiometabolic syndrome. A desynchronization of peripheral clocks in cardiac tissue effect the central metabolic activity surrounding cardiomyocytes, thereby leading to accumulated detrimental intracellular long‐chain fatty acid (LCFA) derivatives resulting in contractile dysfunctions of the heart 61. Linking the occurrence of LCFA derivatives with circadian disruption in noncardiac tissue would contribute to several characteristics of the cardiometabolic syndrome 61.

Evidence is also rising that circadian rhythm and both, the irradiance dosis and the spectral irradiance of light, impact eye growth, or emmetropization. Recent reviews shed light on this complex interaction and the interdependence of the disruption of circadian rhythm on the onset and progression of myopia 112, 113. Clock gene knock‐out animal studies are expected to establish further potential associations between circadian disruptions and human pathologies, which are yet to be covered by epidemiological studies 114.

## 6 COUNTERMEASURES FOR THE ALTERING EFFECTS OF THE BLUE LIGHT

Blue light is currently considered to have the strongest effect in synchronizing human circadian rhythm. Exposure to low levels of blue of light as well as bright light during night or before bed time may disrupt the circadian rhythm with severe general health implications. At the same time blue light exposure during daytime is crucial for the vitality of the organisms. Retinal illumination decreases with age, for example, due to pupillary miosis, which decrease light transmission, especially in the blue part of the light spectrum. Therefore, inadequate low lighting, especially lacking the blue part of the spectrum, may cause circadian rhythm disruptions. This imposes the importance of proper, bright artificial lighting with a more blue‐weighted spectrum during daytime. For example, increasing the blue portion of artificial light may improve performance and learning ability in school kids and employees working indoors, and health will be improved in patients staying at nursing homes or hospitals.

Following the recently accumulated knowledge about circadian rhythm regulation and disruption, it is apparent that there are two key components to keep a healthy circadian system: An increase in the blue portion of the artificial light during daytime should be accompanied with a reduction of the same blue portion of artificial light during the night and evening hours. Electronic manufacturers and software providers already offer a variation of blue light blocking features for displays. While the implementations in general decrease the amount of blue light emitted by the devices, the remaining share of short wavelengths in white light is not accounted for. Importantly, it has been reported that a pure blue light filter has an insufficient effect on melatonin suppression without an accompanied brightness reduction 115. A simple solution that may effectively block and reduce the blue portion of the light spectrum before bedtime are blue light blocking glasses and lenses 116, 117. Wearing blue light blocking eyewear before 118, 119 and during 120 bed time may effectively attenuate LED induced melatonin suppression 121, 122 and thus can potentially facilitate the adaption to new social schedules 123 and reduce sleep disturbances and their consequences among the general population. Further, a reduction of the blue portion in artificial light during nighttime hours could protect shift workers against disorders such as cancer and cardiovascular disorders. Importantly, all solutions should consider the optimal spectral requirements of both, conscious and unconscious photo‐reception.

## CONFLICT OF INTEREST

S.W., M.E., and I.V.I. are researchers at the University of Tuebingen; S.W., P.S., C.L., and I.V.I. are employed by Carl Zeiss Vision International GmbH, manufacturing spectacle lenses and sunglasses. There is no conflict of interest regarding this study.

## AUTHOR CONTRIBUTIONS

S.W. and I.V.I. did the literature study and analysis; S.W., M.E., and I.V.I. wrote the manuscript, P.S. and C.L. designed the illustrations; all reviewed the illustrations and all reviewed critically the manuscript.
